# Prognostic Role of Pre-Treatment [^18^F]FDG PET/CT in Patients with Anaplastic Thyroid Cancer

**DOI:** 10.3390/cancers13164228

**Published:** 2021-08-23

**Authors:** Hyun Jeong Kim, Hang-Seok Chang, Young Hoon Ryu

**Affiliations:** 1 Department of Nuclear Medicine, Yongin Severance Hospital, Yonsei University College of Medicine, Yongin 16995, Korea; khj7564@yuhs.ac; 2 Department of Surgery, Thyroid Cancer Center, Gangnam Severance Hospital, Yonsei University College of Medicine, Seoul 06273, Korea; 3 Department of Nuclear Medicine, Gangnam Severance Hospital, Yonsei University College of Medicine, Seoul 06273, Korea

**Keywords:** anaplastic thyroid carcinoma, [^18^F]FDG PET/CT, prognosis, glycolysis

## Abstract

**Simple Summary:**

This study evaluates the prognostic capability of the ^18^fluorodeoxyglucose ([^18^F]FDG) positron emission tomography (PET)/computed tomography (CT) in patients with anaplastic thyroid cancer (ATC) which can be used as a potential biomarker reflecting glycolysis. ATC is a rare, but highly lethal disease with a one-year overall survival of 20%, and its prognostic factors have rarely been investigated. In this study, survival data correlated with PET/CT derived parameters provide evidence that FDG uptake assessed by PET/CT is a prognostic marker, which may have a clinical impact on the management of patients with ATC.

**Abstract:**

Anaplastic thyroid carcinoma (ATC) is a rare but highly lethal disease. Therefore, its diagnosis at an early stage and a rapid and accurate establishment of a proper treatment strategy is warranted. Tumor glycolysis assessed by ^18^fluorodeoxyglucose ([^18^F]FDG) positron emission tomography (PET)/computed tomography (CT) is predictive of many cancers despite its limited proven applicability to ATC. We investigated the prognostic capability of [^18^F]FDG PET/CT in patients with ATC. Forty patients with ATC were subjected to [^18^F]FDG PET/CT for pre-treatment evaluation. The tumor size and stage, overall survival (OS), and PET parameters, including the maximum standardized uptake value (SUVmax), metabolic tumor volume (MTV), and total lesion glycolysis (TLG) were analyzed. The 1-year OS rate was 17.5% with a mean life expectancy of 7.1 months. Distant metastasis was detected solely using PET/CT in 37.5% of cases. High SUVmax, MTV, and TLG were significantly associated with poor prognosis (*p* < 0.001, *p* = 0.002, and *p* < 0.001, respectively). A significant difference (*p* < 0.001) was observed in OS between patients with a high and low tumor SUVmax. Glucose metabolism assessed by [^18^F]FDG PET/CT was significantly associated with the OS of patients with ATC. PET-derived parameters such as SUVmax, MTV, and TLG are useful prognostic biomarkers for ATC.

## 1. Introduction

Anaplastic thyroid cancer (ATC) is a highly dedifferentiated cancer that retains only a few characteristics of thyroid follicular cells [1,2,3]. ATC is rare, accounting for only 1.3–9.8% of all thyroid cancers [1], but it represents over half of thyroid cancer-related deaths [4]. In addition, ATC is associated with a very poor prognosis [5,6]. The median survival of patients with ATC is approximately 5 months, with a 1-year overall survival (OS) of 20% [1]. All patients with ATC are classified by the American Joint Committee on Cancer (AJCC) TNM system as stage IV [7,8,9], owing to a poor disease prognosis. Therefore, it is imperative to establish a rapid and definitive diagnostic method at the early stage of this cancer because of its substantially rapid growth and aggressiveness. Although there are several management options and recommendations [10], there is no clear consensus or unified treatment of choice. In most studies, larger tumor size, extrathyroidal invasion, and distant metastasis are reported as prognostic factors [11,12], but these factors may not be accurately evaluated prior to surgery. Contradictory results were reported for factors such as age, sex, leukocytosis, and acute symptoms [13,14,15,16,17]. In particular, the usefulness of prognostic factors for ATC before treatment should be further studied.

^18^Fluorodeoxyglucose ([^18^F]FDG) positron emission tomography (PET)/computed tomography (CT) is an imaging modality that visualizes glycolysis using a radioactive biomarker called FDG, a glucose analogue. The [^18^F]FDG PET/CT is a well-established method for the diagnosis, staging, and evaluation of treatment response, recurrence, and prognosis in various malignancies. In particular, the prognostic role of [^18^F]FDG PET/CT has been demonstrated in different cancers, including well-differentiated thyroid cancer [18]. The American Thyroid Association (ATA) guidelines for the management of patients with ATC recommends the use of [^18^F]FDG PET/CT for adjunctive pre-treatment radiological tumor staging [10]; [^18^F]FDG PET/CT is particularly valuable in evaluating metastatic sites and determining tumor resectability. Considering the rarity of ATC, large-scale studies to date have been limited, most have been published as case reports [19,20,21,22,23,24], and only a few studies have investigated the role of [^18^F]FDG PET/CT in ATC [25,26,27]. Bogsrud et al. reported an intense FDG uptake on PET in ATC tumors from 16 subjects. A review article indicated that FDG PET provides a higher sensitivity (66–100%) and specificity (79–90%) than conventional imaging methods in the follow up of medullary thyroid cancer and ATC patients. Poisson et al. reported the benefit of FDG PET/CT in the initial staging in 18 ATC patients. Additionally, they found that the intensity of FDG uptake was suggestive of a poor prognosis. However, the various risk factors of ATC have not yet been explored to determine the prognostic role of [^18^F]FDG PET/CT.

In this study, we aimed to examine the prognostic role of [^18^F]FDG PET/CT in a relatively large cohort of patients with ATC and, thus, demonstrate its applicability as a potential biomarker for ATC prognosis.

## 2. Patients and Methods

### 2.1. Study Subjects

In this retrospective study, we collected data from January 2012 to December 2020 of patients who were at least 20 years of age at the time of ATC diagnosis. The [^18^F]FDG PET/CT was performed prior to any treatment, and the patients were then subjected to standard treatments according to the ATA guideline [10]. The collected clinical data included age, histopathology, lympho-vascular invasion, tumor size, lymph node status, treatment modalities, and survival outcomes. The TNM stage was classified based on the AJCC manual, 8th edition [7,8,9]. Enrolled patients with any other invasive cancer, no electronic medical record, no PET/CT evaluation, any other inflammatory disease (including autoimmune disease and infection), and organ failure were excluded from this study. Patients who underwent PET/CT evaluation at other hospitals were also excluded from this study to maintain uniformity in the PET/CT protocol. The study was approved by the Institutional Review Board (IRB) of the Gangnam Severance Hospital, Korea. The need for informed consent was waived under IRB approval because of the retrospective study design.

### 2.2. PET/CT Imaging Protocol

All patients were fasted for at least 6 h and their blood glucose levels were confirmed to be lower than 140 mg/dL before the administration of [^18^F]FDG (5.5 MBq/kg of body weight) [28]. Sixty minutes after [^18^F]FDG intravenous injection, scanning was performed using a dedicated PET/CT scanner (Biograph mCT, Siemens Healthcare Solutions USA, Inc., Knoxville, TN, USA). Whole body low-dose CT images were obtained for attenuation correction using automatic dose modulation with a reference of 40 mA and 120 kV. PET data were then acquired from the skull base to the proximal thigh for 3 min per bed position in a three-dimensional mode. PET images were reconstructed with the ordered subset expectation maximization algorithm. Maximum intensity projection, cross-sectional views, and fusion images were generated and reviewed. Cross-calibration between the PET and dose calibrator was conducted on a monthly basis.

### 2.3. PET/CT Imaging Analysis

Image analysis was performed using MIM 6.8.10 (MIM software Inc., Cleveland, OH, USA). The PET/CT images were independently reviewed by two experienced nuclear physicians blinded to the imaging studies and clinical and pathologic results. The physicians reached a consensus about the interpretation of the imaging finding.

For semi-quantitative evaluation, the standardized uptake value (SUV) was calculated by measuring the tumor absorption of [^18^F]FDG in the region of interest as follows: SUV = (radioactivity concentration in region of interest (ROI))/(injected dose/patient’s weight (kg)). The volume of interest was drawn over the primary thyroid cancer lesion on the PET/CT images, and the maximum SUV (SUVmax) of the tumor was measured. An iso-contour connecting outline was automatically produced that was equal to or greater than each threshold of 41% of the SUVmax of the primary tumor on each PET/CT axial image. According to the EANM procedure guideline for tumor imaging, the 3D iso-contour at 41% of the maximum pixel value corresponds best with the actual dimensions of the tumor [28]. By adopting the relative threshold of SUVmax as a cutoff, this method can segment the heterogeneous lesions more reliably than by using fixed threshold. Multiple lesions are automatically segmented at once. Metabolic tumor volume (MTV) was calculated by summation of the voxels of each PET/CT slice. Total lesion glycolysis (TLG) was calculated by multiplying the selected PET volume by the average SUV within that volume as follows: TLG = MTV × average SUV.

### 2.4. Statistical Analyses

OS was defined as the time from diagnosis to death from any cause. The data of patients who did not exhibit relevant events were censored at the end of follow-up. Continuous variables between the two groups were compared using the Student’s *t*-test or Mann–Whitney U test. Categorical variables were compared using the chi-square test or Fisher’s exact test. For statistical analysis, all continuous variables were divided into two groups; the specific cut-off values were determined using the Contal and O’ Quigley method [29]. Spearman’s correlation analysis was used to evaluate the relationships between tumor size and PET parameters. Survival curves were obtained by the Kaplan–Meier method, and two-group comparisons were performed using the log-rank test. Univariate and multivariate Cox proportional hazard models were used to identify factors associated with survival outcomes. The variables showing statistically significant differences in the univariate analysis were used in the subsequent multivariate analysis.

Statistical analyses were performed using SPSS version 24 (SPSS Inc., Chicago, IL, USA) software. A value of *p* < 0.05 was considered statistically significant.

## 3. Results

### 3.1. Patient Characteristics

The baseline characteristics and clinicopathologic features of all enrolled patients are shown in Table 1. The analysis of the 40 patients with ATC revealed their mean age to be 67.5 years (range, 41–85), and 50% were female. The 1-year OS was 18%, with a mean OS of 7.1 months, consistent with previous study results [6,30,31]. Distant metastasis was reported in 73% of patients, and the most frequently involved organs were the lung, bone, and brain. Fifteen patients (37.5%) showed distant metastasis only on PET/CT.

### 3.2. PET Parameters

**Figure 1:**
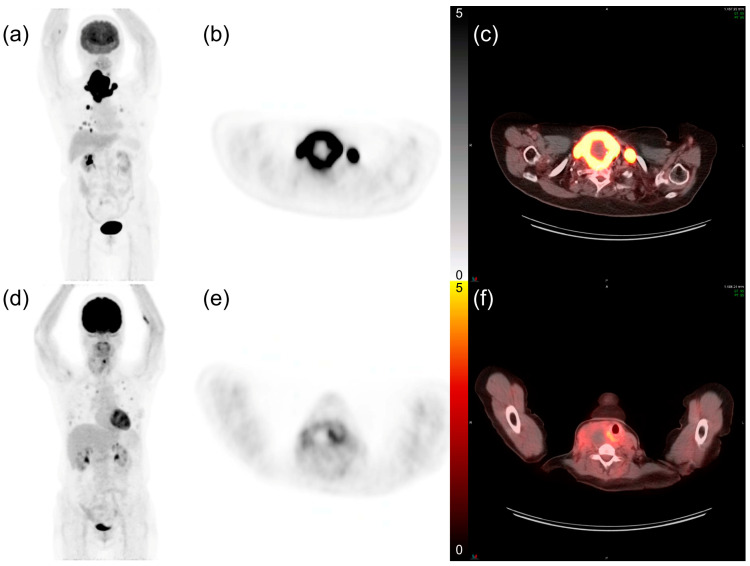
Representative ^18^fluorodeoxyglucose ([^18^F]FDG) positron emission tomography (PET)/computed tomography (CT) images of patients with anaplastic thyroid cancer and lung metastases. (**a**) The maximum intensity projection image of a 74-year-old female patient. (**b**) PET axial and (**c**) fusion axial images show intense FDG uptake in the thyroid tumor. The maximum standardized uptake value (SUVmax) was 35.98, and the patient expired 2.1 months after diagnosis. (**d**–**f**) The maximum intensity projection, PET axial, and fusion axial images of a 63-year-old female patient with a tumor SUVmax of 8.81. Her overall survival time was 8.1 months.

The mean values of SUVmax, MTV, and TLG were 20.7, 88.7, and 843.0, respectively. Tumor size showed a moderate correlation with PET-derived tumor volumes (MTV: ρ = 0.688, TLG: ρ = 0.633, *p* < 0.001 for both) but had no correlation with SUVmax (ρ = 0.278, *p* = 0.083). The optimal cut-off values for SUVmax, MTV, and TLG were 17.97, 59.53 cm^3^, and 604.64 g, respectively, as determined by the Contal and O’ Quigley method [29]. Patients with PET parameters greater than or equal to the cut-off value were categorized as the high group, and patients with parameters less than the cut-off value were categorized as the low group. Among all patients, 21 (52.5%) belonged to the high SUVmax group, 23 (57.5%) in the high MTV group, and 21 (52.5%) in the high TLG group. Figure 1 shows representative [^18^F]FDG PET/CT images of patients with ATC exhibiting a high and low FDG uptake.

### 3.3. Kaplan–Meier Survival Analysis

**Figure 2:**
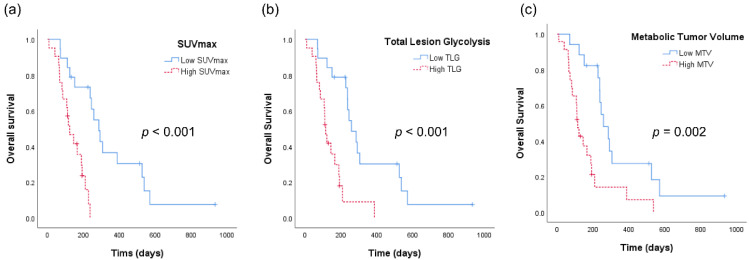
Kaplan–Meier analyses of patients with anaplastic thyroid cancer. (**a**) Overall survival curves according to the maximum standardized uptake value (SUVmax) of tumor. (**b**) Overall survival curves according to total lesion glycolysis (TLG). (**c**) Overall survival curves according to metabolic tumor volume (MTV).

Figure 2 shows the Kaplan–Meier analyses of patients with ATC according to the aforementioned PET-derived parameters. The OS was 347 days for patients with low SUVmax as compared with an OS of 135 days for patients with high SUVmax (*p* < 0.001, Figure 2a). Similarly, patients with low TLG had a significantly longer OS than those with high TLG (OS 343 vs. 142 days, *p* < 0.001, Figure 2b). OS also showed a statistically significant difference with respect to MTV (OS 349 vs. 163 days, *p* = 0.001).

### 3.4. Univariate and Multivariate Analyses

The statistically significant variables for predicting OS in univariate and multivariate analyses are shown in Table 2. No statistical difference was observed in the baseline characteristics, including age, association with differentiated cancer, capsular invasion, lymph node metastasis, and treatment modalities. According to the optimal cut-off value of tumor size, 24 (60.0%) patients belonged to the large tumor group. Tumor size, local extension, distant metastasis, SUVmax, MTV, and TLG were significant prognostic factors in the univariate analysis (*p* = 0.007, *p* = 0.010, *p* = 0.031, *p* < 0.001, *p* = 0.002, and *p* < 0.001, respectively). As a significant correlation was observed between MTV and TLG (r = 0.927, *p* < 0.001), MTV and TLG models were separately analyzed. In the multivariate analysis, SUVmax independently predicted OS with a hazard ratio (HR) of 5.105 (*p* = 0.010) in the MTV model and 4.673 (*p* = 0.017) in the TLG model.

## 4. Discussion

In this study, we evaluated the applicability of [^18^F]FDG PET/CT as a prognostic marker in patients with ATC.

Given the rarity of ATC and the short-life expectancy of affected patients, obtaining sufficient quantities of data from patients with ATC was difficult. Therefore, it was challenging to advance our understanding of the factors that may influence treatment responses and the resulting survival rates. In the present study, survival datasets from 40 patients with ATC were obtained and analyzed. The SUVmax of the tumor was a significant prognostic factor in the multivariate analysis of OS. To the best of our knowledge, this is the first study to identify the prognostic role of [^18^F]FDG PET/CT by analyzing PET-derived parameters along with various risk factors.

The [^18^F]FDG PET-CT is a molecular imaging technique that can visualize glucose consumption non-invasively in vivo using [^18^F]FDG, a glucose analogue. It is widely used to reveal tumor glucose metabolism in patients with various cancers [32,33,34,35,36]. Especially in the preoperative setting, it is the most efficient and useful method for demonstrating glucose consumption. The 2012 ATA guideline [10] suggests that [^18^F]FDG PET/CT can detect GLUT1 glucose transporter overexpression in ATC cells, as it results in a significant increase in FDG uptake [37]. The NCCN guideline also recommends PET/CT scans to accurately stage the disease in patients with ATC [38]. Previous studies have reported the significant role of FDG PET or PET/CT in ATC for staging, follow up, detecting residual or metastatic disease, and assessing therapeutic response [23,25,26]. In these studies, FDG PET scan findings altered the management recommendations in 25–50% of patients. Poisson et al. were the first to show that FDG uptake is a significant prognostic factor for survival in patients with ATC. However, this study, which was conducted a decade ago, included only 18 patients and did not consider the effects of other prognostic factors [27].

In the present study, distant metastasis was detected solely by PET/CT in 15 patients (37.5%); the locations of metastasis were found to be the lungs (7 patients), bones (6 patients), distant lymph nodes (5 patients), and brain (1 patient). In particular, [^18^F]FDG PET/CT was effective in identifying bone lesions, which may not be visible in CT images. PET/CT is a preoperative non-invasive imaging modality that can be used for a whole body scan in a single examination. In 4 out of 15 patients, distant metastases in more than two organs were solely detected by PET/CT.

In the univariate analysis, PET parameters such as SUVmax, MTV, and TLG (*p* < 0.001, *p* = 0.002, and *p* < 0.001, respectively) were found to be more reliable and significant prognostic factors than tumor size (*p* = 0.007) or stage (*p* = 0.031). This finding has a great clinical impact because FDG uptake, which reflects tumor glycolysis, can be a more efficient prognostic indicator than the previously known risk factors of ATC; neither the type of treatment modality nor R0 resection showed statistical significance in the prognosis. However, it should be considered that a unified treatment method for ATC has not yet been proposed and that the condition of the patient varies depending on the location of occurrence even if the tumor burden is similar. Although sex as a risk factor is still controversial, a multivariate analysis in a previous study showed that old age, node invasion, capsular invasion, and the female sex were poor prognostic factors [39]. Tumor size and distant metastasis is a significant predictor for OS in univariate analysis (*p* = 0.007, and 0.031), but it was not significant in a multivariate analysis (*p* = 0.163, and 0.063). This discrepancy may be due to multicollinearity and the relatively small number of sample size. Considering the rapidly progressing characteristics of ATC, the disease can be dismal even if there is no distant metastasis at the time of diagnosis. In addition, if there is a mass effect such as an airway or vessel invasion, it could be lethal independently. In the multivariate analysis, MTV and TLG were not identified as statistically significant factors for OS in each model. There were several hypotheses that could explain this observation, including a statistical correlation between SUVmax and volume-based parameters, low FDG uptake in the tumor owing to necrosis, anatomical specificity such as a possible catastrophic airway compromise, and the presence of lung metastasis with multiple nodules even if the volume is small. Due to the multicollinearity and small number of samples, the statistical analysis according to the type of surgery and T stage was limited. Instead, the analysis using a cut-off value of tumor size and the presence of a local extension were performed in the Cox proportional hazard model analysis.

The [^18^F]FDG PET/CT findings were expected to have a direct impact on the management of patients with ATC because the data suggested that patients with a high tumor FDG uptake had a poor prognosis, and such [^18^F]FDG PET/CT findings would allow for a more aggressive treatment to be recommended in the early stages of disease. By additionally detecting metastases with pre-treatment PET/CT, an appropriate treatment plan can be established involving surgery, radiation therapy, and/or chemotherapy. In addition, by providing accurate information to patients with a low life expectancy or to caregivers, suitable assistance such as palliative or terminal health care can be provided.

The present study had some limitations. First, this study was retrospectively performed at a single institution. Second, as ATC itself has a wide physiopathological spectrum and there is no universal treatment method, selection bias could have been reduced through a more refined and homogenous patient group. Third, we measured SUV, a clinically accepted parameter for PET studies, to quantify FDG uptake. SUV itself can be influenced by multiple factors such as plasma glucose and insulin levels, although this is an inevitable point that all the researches using SUV face. Thus, further validation is warranted through prospective multicenter studies with a larger number of patients in the future. Despite these limitations, this study clearly identified the applicability of tumor FDG uptake to predict the outcomes of patients with ATC.

This study revealed that PET-derived parameters can play a prognostic role in ATC and can have a great clinical impact. The diagnosis and initial evaluation of ATC are still challenging and critical [40,41]. In cancer cells, GLUT1 expression, tumor proliferation, and the resulting glucose uptake reflect tumor aggressiveness. We believe that future follow-up studies on the correlation with radiomics or cellular molecular marker related to FDG PET/CT could solidify the importance of tumor glucose metabolism and resulting impacts on the prognosis in patients with ATC. Pre-treatment [^18^F]FDG PET/CT in patients with ATC to determine the extent of disease as well as for prognosis can facilitate multidisciplinary team engagement and coordination for selected patients. In the future, the application of [^18^F]FDG PET/CT needs to be particularly specified in the ATC guidelines.

## 5. Conclusions

Glucose metabolism of the tumor as assessed by [^18^F]FDG PET/CT was found to be related with the outcomes of patients with ATC. PET/CT was excellent for detecting distant metastasis, and PET-derived parameters such as SUVmax, MTV, and TLG were significantly associated with the OS of patients with ATC. Especially, SUVmax independently predicted OS in the multivariate analysis. FDG uptake assessed by pre-treatment PET/CT is a significant prognostic marker, which may have a clinical impact on the management of ATC patients.

## Figures and Tables

**Table 1:** Patient characteristics.

Patient Characteristics	Value	Percent
Total number of patients	40	100%
Sex (female)	20	50%
Age (year, mean)	67.5 ± 9.9	
Tumor size (cm, mean)	5.3 ± 2.2	
Stage *		
IVA	4	10%
IVB	7	17%
IVC	29	73%
Distant metastasis		
Distant node	22	55%
Lung	26	65%
Bone	7	18%
Brain	2	5%
Treatment		
No treatment	1	3%
Surgery	13	33%
CTx	34	85%
RTx	31	78%
CTx + RTx	22	55%
Multimodality Tx	35	88%
Survival		
1-year OS	17.5%	
Mean OS (month)	7.1	
PET parameters (mean)		
SUVmax	20.7 ± 12.7	
SUVpeak	17.9 ± 10.1	
MTV	88.7 ± 82.1	
TLG	843.0 ± 940.7	

CTx, chemotherapy; RTx, radiation therapy; OS, overall survival; PET, positron emission tomography; SUVmax, maximum standardized uptake value; MTV, metabolic tumor volume; TLG, total lesion glycolysis. * AJCC stage was determined based on the 8th edition.

**Table 2:** Univariate and multivariate analyses of overall survival.

	Univariate Analysis		Multivariate Analysis (MTV Model)		Multivariate Analysis (TLG Model)	
**Patient Characteristics**	**HRs (95% CIs)**	***p*** **Value**	**HRs (95% CIs)**	***p*** **Value**	**HRs (95% CIs)**	***p*** **Value**
Sex (Female)	2.929 (1.337–6.417)	0.006	1.032 (0.417–2.555)	0.945	1.038 (0.419–2.570)	0.936
Age (>64 years)	1.978 (0.937–4.173)	0.074				
Tumor characteristics						
Tumor size (>4.7 cm)	2.946 (1.344–6.460)	0.007	1.854 (0.780–4.410)	0.163	1.844 (0.796–4.271)	0.153
Association with differentiated cancer	0.543 (0.237–1.245)	0.149				
Capsular invasion	1.127 (0.309–4.108)	0.856				
Local extension	4.999 (1.479–16.897)	0.010	3.720 (0.946–14.634)	0.060	3.343 (0.846–13.203)	0.085
Lymph node metastasis	1.931 (0.670–5.562)	0.223				
Distant metastasis	2.270 (1.078–4.779)	0.031	2.222 (0.957–5.160)	0.063	2.276 (0.997–5.194)	0.051
PET parameters						
High SUVmax	6.558 (2.319–18.546)	<0.001	5.105 (1.489–17.508)	0.010	4.673 (1.325–16.484)	0.017
High MTV	3.207 (1.516–6.781)	0.002	1.309 (0.474–3.611)	0.603		
High TLG	4.239 (1.903–9.441)	<0.001			1.515 (0.518–4.425)	0.448
Treatment						
Surgery	0.459 (0.207–1.015)	0.054				
R1 resection	3.198 (0.283–36.091)	0.347				
CTx	1.360 (0.411–4.500)	0.615				
RTx	0.891 (0.339–2.341)	0.815				
Multimodality Tx	0.954 (0.363–2.508)	0.924				

PET, positron emission tomography; SUVmax, maximum standardized uptake value; MTV, metabolic tumor volume; TLG, total lesion glycolysis; CTx, chemotherapy; RTx, radiation therapy; Tx, therapy; HR, hazard ratio; CI, confidence interval.

## Data Availability

The data presented in this study are available on request from the corresponding author. The data are not publicly available due to confidentiality reasons.
